# Improved Diabetes Care Management Through a Text-Message Intervention for Low-Income Patients: Mixed-Methods Pilot Study

**DOI:** 10.2196/diabetes.8645

**Published:** 2018-10-30

**Authors:** Jessica L Watterson, Hector P Rodriguez, Stephen M Shortell, Adrian Aguilera

**Affiliations:** 1 Center for Healthcare Organizational and Innovation Research School of Public Health University of California, Berkeley Berkeley, CA United States; 2 School of Social Welfare University of California, Berkeley Berkeley, CA United States; 3 Department of Psychiatry University of California, San Francisco San Francisco, CA United States

**Keywords:** diabetes mellitus, type 2, text messaging, telemedicine, health education, qualitative research, poverty, Hispanic Americans

## Abstract

**Background:**

Diabetes is a major contributor to global death and disability. Text-messaging interventions hold promise for improving diabetes outcomes through better knowledge and self-management.

**Objective:**

The aim of this study was to examine the implementation and impact of a diabetes text-messaging program targeted primarily for low-income Latino patients receiving care at 2 federally qualified health centers (FQHCs).

**Methods:**

A mixed-methods, quasi-experimental research design was employed for this pilot study. A total of 50 Spanish or English-speaking adult patients with diabetes attending 2 FQHC sites in Los Angeles from September 2015 to February 2016 were enrolled in a 12-week, bidirectional text-messaging program. A comparison group (n=160) was constructed from unexposed, eligible patients. Demographic data and pre/post clinical indicators were compared for both the groups. Propensity score weighting was used to reduce selection bias, and over-time differences in clinical outcomes between groups were estimated using individual fixed-effects regression models. Population-averaged linear models were estimated to assess differential effects of patient engagement on each clinical indicator among the intervention participants. A sample of intervention patients (n=11) and all implementing staff (n=8) were interviewed about their experiences with the program. Qualitative data were transcribed, translated, and analyzed to identify common themes.

**Results:**

The intervention group had a mean glycated hemoglobin (HbA_1c_) reduction of 0.4 points at follow-up, relative to the comparison group (*P*=.06). Patients who were more highly engaged with the program (response rate ≥median of 64.5%) experienced a 2.2 point reduction in HbA_1c_, relative to patients who were less engaged, controlling for demographic characteristics (*P*<.001). Qualitative analyses revealed that many participants felt supported, as though “someone was worrying about [their] health.” Participants also cited learning new information, setting new goals, and receiving helpful reminders. Staff and patients highlighted strategies to improve the program, including incorporating patient responses into in-person clinical care and tailoring the messages to patient knowledge.

**Conclusions:**

A diabetes text-messaging program provided instrumental and emotional support for participants and may have contributed to clinically meaningful improvements in HbA_1c_. Patients who were more engaged demonstrated greater improvement. Program improvements, such as linkages to clinical care, hold potential for improving patient engagement and ultimately, improving clinical outcomes.

## Introduction

### Background

An estimated 29.1 million people have diabetes in the United States [1] and over 2.3 million adults in California report being diagnosed [2]. As one of the most common chronic illnesses, diabetes leads to an estimated US $245 billion in economic costs annually and doubles the risk of death for those affected [1]. Furthermore, the prevalence of diabetes among Latinos is almost double than that of non-Latino whites, and rates of diabetes are also much higher among people with lower incomes and education [3]. In addition to higher rates of disease, evidence suggests that low-income patients also experience worse complications resulting from diabetes [4].

### Objectives

Text-messaging interventions for people with diabetes hold promise for improving patient satisfaction and intermediate health outcomes through better knowledge and self-management. In particular, there is evidence that text-messaging programs can improve glycated hemoglobin (HbA_1c_ levels in people with diabetes [5-8]. Following participation in these types of programs, patients have reported high levels of satisfaction and changes to their diet and other behaviors, which should lead to improved management of their diabetes [9-11].

Despite the benefits of these interventions in broader populations, studies have found that patient engagement and the resulting health effects can be worse for people who are nonwhite, have lower literacy, and/or are older [12-14]. However, there is potential to improve the effectiveness of diabetes interventions through culturally sensitive adaptations [15]. This fact, coupled with the higher prevalence of diabetes among Latino and low-income populations, highlights the importance of targeting the interventions to Latino populations and examining their impact on care. To contribute to this aim, *we studied the impact of a pilot diabetes text-messaging program targeted primarily for low-income Latino patients, receiving care in federally qualified health centers (FQHCs)*.

In addition to assessing the pilot program’s impact, *we also examined implementation barriers and facilitators through interviews with patients and staff*. Identifying the operational- and patient-factors influencing implementation effectiveness can inform the effective scale-up of similar text-messaging interventions in other clinics and health systems caring for patients who are Latino and/or have low income.

## Methods

### Overview

This study employed a mixed-methods, quasi-experimental design to examine the effectiveness and implementation of a pilot 3-month short message service intervention for adult patients with diabetes, which sent automated, interactive text messages focused on diabetes self-management. Quantitative data included program and clinical indicators, and qualitative data included semistructured interviews of patient participants and clinic staff.

### Setting

Participants (n=50) were Spanish- (n=33) or English-speaking (n=17) adult patients with diabetes attending 2 sites of ChapCare, an FQHC in Los Angeles, from September 2015 to February 2016. From October to December 2015, enrollment in the pilot intervention was offered to all adult patients with type 2 diabetes with an HbA_1c_ value above 8.5% that presented for an appointment at either of the 2 participating ChapCare clinics. The HbA_1c_ cutoff was suggested by the clinical staff, as they felt these patients might benefit most from the intervention. However, in January and February 2016, due to limited enrollment, patient eligibility was expanded to include all adult patients with type 2 diabetes who presented for an appointment, until intervention group enrollment reached 50 participants. Enrollment procedures and staff involved in the intervention were determined by the clinic administration and are examined in the implementation component of this study. Clinic front desk staff identified eligible patients with diabetes from a preprinted list when they checked in for their appointment. The patient was then referred to an AmeriCorps volunteer, who explained the text-messaging program and offered to help them enroll. To enroll, patients had to have their own mobile phone with text-messaging capabilities. Out of 65 patients who were approached, 77% (50/65) enrolled in the text-messaging program. For the 15 patients who declined to enroll in the intervention, the following reasons were given: no mobile phone (27%, 4/15), not comfortable with text messaging (20%, 3/15), not interested in receiving health information via text (40%, 6/15), and already comfortable with managing their diabetes (13%, 2/15). No compensation was given to participants for participating in the text-messaging program. The sample size of 50 intervention participants and the follow-up period was selected based on earlier studies of text-messaging programs for patients with diabetes that examined HbA_1c_, body mass index (BMI), and blood pressure (BP) as outcomes [5] and to limit disruption to the pilot clinics.

A comparison group (n=160) of adult patients with type 2 diabetes was constructed through chart review. All patients with type 2 diabetes who attended the clinics during the study period but were unexposed to the intervention and attended a follow-up visit before August 2017 were eligible for inclusion in the comparison group.

### Intervention

**Figure 1:**
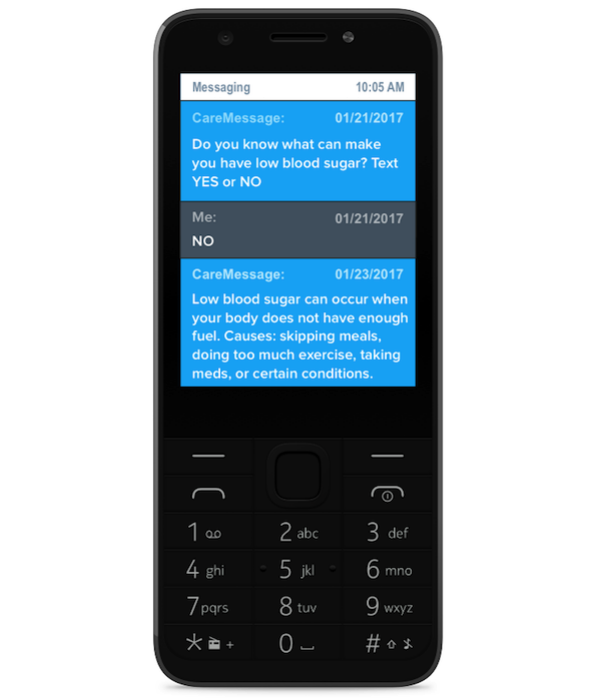
Sample text message.

The text-messaging intervention was designed for adults with diabetes using a proprietary platform from CareMessage, a nonprofit organization that designs mobile health tools. The 12-week intervention consisted of 3 to 4 educational text messages per week in either Spanish or English, depending on the participants’ preference. Most of the messages were bidirectional: 31% were multiple-choice and 24% asked yes/no or true/false questions, similar to the example message in Figure 1. If a participant answered incorrectly, they would receive a gentle response with the correct answer. If the participant answered correctly, they received a response affirming that their answer was correct. The remaining 45% of messages were unidirectional (eg, a health tip or reminder).

The program was targeted at low-income patients, and the Spanish-language version was further targeted at Latino patients. The Spanish program was not a direct translation of the English program, but instead it was developed from the beginning of the program’s conceptual design stages in Spanish. The development of both programs was informed by observing patients in one-on-one and group education sessions conducted by CareMessage in community clinics. In addition, CareMessage conducted focus groups with patients with diabetes after they received the messages as part of a 3-month feasibility study in San Francisco in 2014. Following this product development research, the messages were targeted to address participants’ concerns and culture.

For example, Spanish-speaking patients more often discussed how family and traditional foods sometimes made it difficult to change their behavior; therefore, the Spanish messages were adapted to address this topic and to include foods that may be prevalent in Latino populations. Some messages were also adapted to incorporate income level into recommendations for exercise and disease management. For example, patients expressed concerns about being able to afford test strips and therefore, with guidance from a physician, the message was adapted to state they could potentially skip a day so they did not run out of test strips as quickly.

The messages address 10 overall themes: understanding diabetes, medication adherence, nutrition, exercise, mental health, resources, managing blood sugar levels, ABCs (A_1c_, BP, and cholesterol), foot care, and annual exams (eye, kidney, and dental). The messages were developed using the American Diabetes Association guidelines for disease self-management along with input from the health care providers at implementing clinics and oversight of the staff physician at CareMessage. The average-grade reading level of the unique messages in the program is 6.2, according to the Flesch-Kincaid Grade Level test [16].

### Quantitative Program Data

#### Collection

At baseline, intervention participants answered 5 questions about diabetes-related emotional distress, Problem Areas in Diabetes questionnaire (PAID-5) in person with the AmeriCorps member, right after registering for the text-messaging program [17]. Throughout the 12-week program, the text-messaging platform recorded patient response rates (calculated by dividing the number of valid responses from the patient by the total number of questions requiring a response, multiplied by 100). At the end of the program, the follow-up PAID-5 questions and a user satisfaction survey were administered via text message. Demographic and clinical data were extracted by chart review from Chapcare’s electronic health record. These data included pre- and postintervention measures of HbA_1c_, BMI, and BP. Premeasurements and demographics were taken from visits to the clinic immediately before the start of the intervention. A single postmeasurement was taken for each patient whenever they presented for their next follow-up visit, sometime between the end of the intervention and up to 1 year from the study commencement date (ie, between February and September 2016). These data from charts were merged with the program data for the intervention group and deidentified before being shared with the research team. A deidentified dataset with the same demographic and clinical measures for the comparison group was also provided to the research team, and the 2 datasets were integrated for analyses.

#### Analysis

First, descriptive statistics were examined for all study variables. This included mean, median, and SD for all continuous variables and frequencies, proportions, and CIs for all categorical variables. Baseline characteristics were compared between the intervention and comparison groups and between patients with missing and complete datasets using chi-square tests for categorical variables and 2-sample *t* tests with unequal variances for continuous variables. The analysis was then restricted to patients with complete baseline and follow-up measures of the dependent variables (HbA_1c_, BP, and BMI). This resulted in listwise deletion of 25 observations (12 from intervention group and 13 from comparison group). Next, propensity score weights were calculated using gender, age, race/ethnicity, and baseline HbA_1c_. A further 8 observations (all from the comparison group) were dropped because of missing data on race/ethnicity, which are needed to calculate the propensity score. Changes in clinical outcomes were compared between groups using individual fixed-effects linear regression models with an ordinary least squares estimator. A sensitivity analysis was run with multiple imputations to handle missing data on the independent variable of race/ethnicity for 8 observations (all from the comparison group). The chained equations method was used, under the missing-at-random assumption, to generate 10 imputed datasets. Propensity score weighting was then conducted for each of the 10 imputed datasets, and the results were combined in the subsequent analysis using Rubin combination rules [18]. Next, the individual fixed-effects linear regression models were run, and results were compared with the main analysis.

The final set of analyses was conducted on the data from the intervention group only. To examine associations between clinical indicators by time-invariant characteristics among intervention participants, population-averaged linear models were estimated with generalized estimating equations. These models facilitated the examination of differential effects of patient engagement on improvements in clinical outcomes among the intervention participants.

An additional post hoc regression model was run to examine any associations between satisfaction with the program and personal characteristics, including patient engagement among the intervention participants.

All models were run with cluster robust SEs to correct for heteroscedasticity and were clustered by patient identity document (to account for the fact that pre/post observations were clustered under each patient). Analyses were conducted with StataSE v.13 (StataCorp).

### Qualitative Program Data

#### Collection

All intervention participants were invited to complete a phone interview to provide feedback on the program in March 2016 (depending on when they enrolled, this ranged from right after the end of the messages to up to 8 weeks after the end of the messages). A total of 11 out of the 50 (22%) participants agreed to be interviewed in their primary language, either Spanish (n=6) or English (n=5). In addition, all 8 staff members participating in the implementation of the program were invited to participate in a phone interview to provide feedback on the program implementation in March 2016 (after enrollment ended in their clinics), and all agreed to participate.

Verbal consent was obtained from all interview participants, and all of them received a gift card as a token of appreciation for their time. Structured interviews lasted up to 45 min and were recorded with the participants’ permission. Interviews were conducted via phone by a researcher in either English or Spanish, depending on the participants’ preference. The structured question guide, with probes, was used to facilitate discussion. The interview guide for participants asked questions aimed at understanding their experience with the program, such as “Describe your first encounter with the text messages. What did you think?” The staff interview guide focused on implementation of the program and asked questions such as “How easy or difficult has it been to incorporate CareMessage into your workflow?” The full interview guides in English are available in Multimedia Appendix 1. The university’s review board for research with human subjects approved the research study.

#### Analysis

Interview recordings were professionally transcribed and when applicable, were translated from Spanish to English by a bilingual member of the research team. A preliminary codebook was developed by 1 researcher, drawing upon the existing literature on text messages for health as well as the Health Belief Model [19] and related theory. Coding of all patient interviews was then performed by 2 researchers, using ATLAS.ti software (ATLAS.ti Scientific Software Development GmbH). The coding process was iterative, and the codebook grew throughout the analysis as additional codes were added based on the data. If a quote emerged that did not fit the preliminary codebook, a new relevant code was generated and discussed with the other researcher. For example, 1 patient explained that they thought the messages were automatically generated but sounded like they came from a person. Preliminary codes only included “automatically generated” or “from a person,” so this data point generated a new code to accommodate this finding. Coding of staff interviews was performed by 1 researcher. After coding was complete, common themes were identified. New concepts and themes were discussed among the research team until the codebook was finalized and all themes had been identified.

## Results

### Quantitative Results

Though demographic (Table 1) characteristics of patients in both the intervention and comparison groups were mostly comparable at baseline, there were some nonstatistically significant differences between groups. Among the groups, 55.7% (117/210) of patients were primarily Spanish speaking. In addition, 69.0% (145/210) of participants were of Hispanic or Latino ethnicity. Participants ranged widely in age, and there were more female participants (62.4%, 131/210) than males (37.6%, 79/210) in both groups. There was a higher proportion of English speakers and females in the comparison group than the intervention group; however, the differences were not statistically significant. Propensity score weighting resolves imbalances of unweighted analyses and helped to further reduce overall mean bias on these observable characteristics by 5.2% and overall median bias by 7.9%.

Most participants (86%, 43/50) in the intervention group responded to at least 1 question with a valid answer (ie, 1 of the multiple-choice options provided). Participants received an average of 31.8 (interquartile range 28-35) questions requiring an answer over the course of the program. The average number of days that participants were enrolled in the program was 79.5 (SD 11.4), with only 3 participants leaving the program before 80 days. No reason was given when participants withdrew—they only had to text the word “STOP” or to tell the clinic staff member who enrolled them that they wished to stop receiving messages. The overall mean response rate was 57.1% (calculated by dividing the number of valid responses from the patient by the total number of questions requiring a response, multiplied by 100), but it varied widely (SD 33.2%).

Table 2 outlines self-reported health indicators from participants in the intervention group, including the levels of diabetes-related distress (PAID-5) that participants were experiencing at baseline and follow-up (after the text-messaging program). Response rates to the follow-up PAID-5 text-message survey were relatively low, ranging from 12% to 54% (depending on the question), and therefore, may not be representative of all participants’ experiences. Most participants reported being in fair or poor health (80%, 39/49) at baseline. In addition, most participants indicated some problems with feeling scared about living with diabetes (54%, 27/50), feeling depressed about living with diabetes (52%, 26/50), worrying about the future (74%, 37/50), and other measures of diabetes-related distress at baseline.

Following propensity score weighting, clinical indicators of patients (Table 3) in the intervention and comparison groups were similar at baseline. The intervention group had slightly higher HbA_1c_ at baseline than the comparison group (8.7 vs 8.0), but the difference was not statistically significant (*P*=.07).

To check for systematic differences between patients who were excluded due to missing outcome data (n=25), their baseline demographics and clinical indicators were compared with the other patients in their respective group using chi-square tests for categorical data and *t* tests for continuous data (results not shown in table). No statistically significant differences in age, gender, race/ethnicity, smoking status, baseline HbA_1c_, baseline diastolic BP, or baseline BMI were detected. However, excluded patients were statistically significantly more likely to speak English than those remaining in both the intervention (58% vs 26%, *P*=.04) and comparison groups (77% vs 45%, *P*=.03). In addition, patients excluded from the comparison group had statistically significantly higher baseline systolic BP than those remaining in the comparison group (144.2 vs 125.8, *P*=.01).

**Table 1:** Baseline characteristics of participants.

Variable		Unadjusted		*P*^a^ value	Before PSW^b^		After PSW, comparison mean (n=140)	*P*^c^ value
		Intervention frequency (n=50), n (%)	Comparison frequency (n=160), n (%)		Intervention mean (n=38)	Comparison mean (n=140)		
**Clinic**				<.001				<.001
	Site 1	18 (36)	21 (13.1)	—^d^	0.37	0.12	0.12	—
	Site 2	32 (64)	139 (86.9)	—	—	—	—	—
**Age group, in years**				.53				—
	18-44	12 (24)	28 (17.5)	—	0.29	0.17	0.19	.19
	45-54	16 (32)	50 (31.2)	—	0.32	0.31	0.32	<.001
	55-64	22 (44)	82 (51.3)	—	0.39	0.52	0.49	.29
**Gender**				.16				.67
	Male	23 (46)	56 (35.0)	—	0.42	0.35	0.38	—
	Female	27 (54)	104 (65.0)	—	—	—	—	—
**Primary language**				.09				.08
	English	17 (34)	76 (47.5)	—	0.26	0.45	0.42	—
	Spanish	33 (66)	84 (52.5)	—	—	—	—	—
**Race and ethnicity**				.43				
	Hispanic or Latino	37 (74)	108 (67.5)	—	0.79	0.69	0.74	.50
	White	5 (10)	18 (11.3)	—	0.05	0.12	0.10	.32
	Other	8 (16)	26 (16.3)	—	0.16	0.16	0.16	>.99
	Missing	0 (0)	8 (5.0)	—	—	—	—	—
**Smoking status**				.53				.34
	Current nonsmoker	48 (96)	147 (91.9)	—	0.03	0.07	0.07	—
	Current smoker	2 (4)	13 (8.1)	—	—	—	—	—

^a^*P* values are for chi-square tests or Fisher exact test where cell frequencies are less than 5.

^b^PSW: propensity score weighting.

^c^*P* values are for *t* tests.

^d^Not applicable.

**Table 2:** Self-reported health indicators of intervention group.

Indicators		Baseline, n (%)	Follow-up, n (%)
**Overall health**		**N=49**	
	Poor	6 (12)	—^a^
	Fair	33 (67)	—
	Good	8 (16)	—
	Very good	2 (4)	—
	Excellent	0 (0)	—
**Feeling scared when I think about living with diabetes**		**N=50**	**N=27**
	Not a problem/minor problem	33 (46)	12 (44)
	Moderate/somewhat serious/serious problem	27 (54)	15 (56)
**Feeling depressed when I think about living with diabetes**		**N=50**	**N=15**
	Not a problem/minor problem	24 (48)	7 (47)
	Moderate/somewhat serious/serious problem	26 (52)	8 (53)
**Worrying about the future and possible serious complications**		**N=50**	**N=11**
	Not a problem/minor problem	13 (26)	3 (27)
	Moderate/somewhat serious/serious problem	37 (74)	8 (73)
**Diabetes takes up too much of my mental and physical energy**		**N=50**	**N=11**
	Not a problem/minor problem	19 (38)	4 (36)
	Moderate/somewhat serious/serious problem	31 (62)	7 (64)
**Coping with complications of diabetes**		**N=50**	**N=15**
	Not a problem/minor problem	19 (38)	4 (27)
	Moderate/somewhat serious/serious problem	31 (62)	11 (73)
**In the past week, how many times have you had a low blood sugar reaction (sweating, weakness, anxiety, trembling, hunger, or headache)?**		**N=50**	**N=14**
	0	20 (40)	4 (29)
	1-3	26 (52)	8 (57)
	4 or more	4 (8)	2 (14)

^a^Not applicable.

**Table 3:** Propensity score weighted diabetes clinical indicators at baseline and follow-up.

Variable	Baseline			Follow-up			Mean difference	
	Intervention (n=38), mean	Comparison (n=140), mean	*P*^a^ value	Intervention (n=38), mean	Comparison (n=140), mean	*P*^a^ value	Intervention (n=38)	Comparison (n=140)
Glycated hemoglobin	8.7	8.0	.07	8.4	8.3	.63	−0.3	0.3
Systolic blood pressure	124.2	124.6	.88	126.6	127.1	.89	2.4	2.5
Diastolic blood pressure	77.1	77.3	.91	77.5	75.4	.23	0.4	−1.9
Body mass index	32.7	33.5	.59	32.4	33.3	.53	−0.3	−0.2

^a^*P* values are for two-tailed, 2-sample *t* tests.

**Figure 2:**
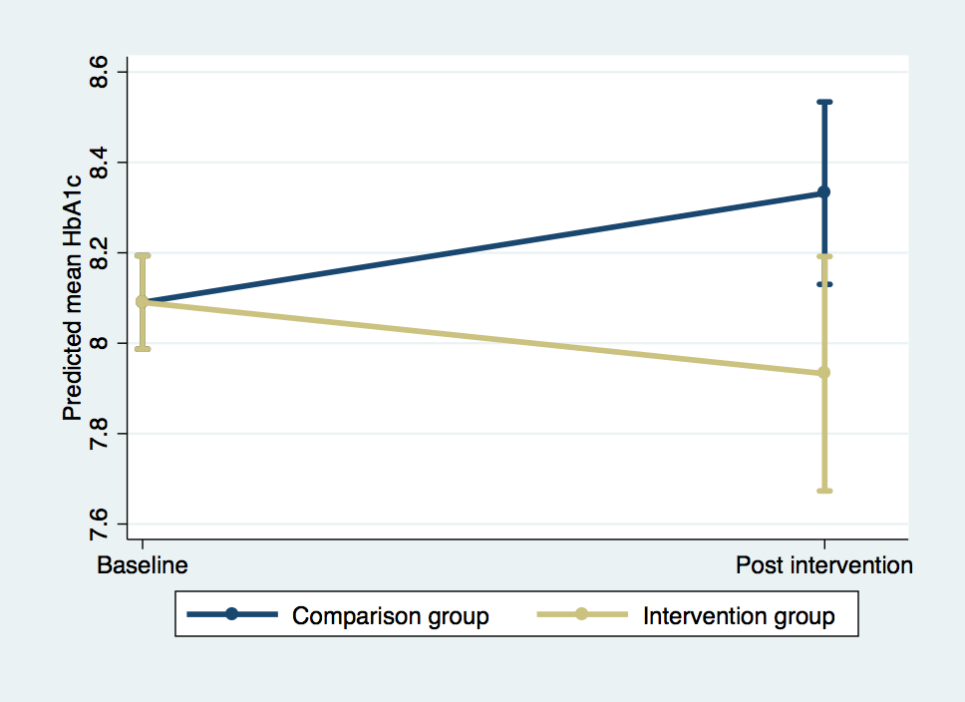
Comparison of adjusted predictions of mean glycated hemoglobin (HbA_1c_) with 95% CIs.

Individual fixed-effects linear regression models (Table 4) on the propensity score weighted data indicate that the intervention group had an average estimated reduction in HbA_1c_ of 0.40 points at follow-up, relative to the comparison group (*P*=.06). This comparison is illustrated graphically in Figure 2. No significant differential reductions were found for BP or BMI. The sensitivity analysis, using multiple imputations for missing independent variables followed by propensity score weighting, produced similar results to the main analysis (results not shown in table). However, baseline balance between groups was not achieved, and bias increased on some variables following propensity score weighting.

**Table 4:** Comparison of change in clinical indicators from baseline to follow-up between intervention and comparison groups.

Coefficient	Glycated hemoglobin (n=185)			Systolic BP^a^ (n=185)			Diastolic BP (n=185)			Body mass index (n=185)		
	Estimate	SE^b^	*P* value	Estimate	SE^b^	*P* value	Estimate	SE^b^	*P* value	Estimate	SE^b^	*P* value
Constant	8.09	0.05	<.001	124.93	0.84	<.001	76.86	0.63	<.001	32.96	0.08	<.001
Time	0.24	0.13	.06	2.43	1.29	.06	−1.89	0.98	.06	−0.13	0.15	.39
Intervention group × time	−0.40	0.21	.06	−1.02	3.38	.76	3.17	2.53	.21	−0.13	0.33	.70
Within-cluster SD	0.95	—^c^	—	3.37	—	—	2.86	—	—	1.08	—	—

^a^BP: blood pressure.

^b^Cluster-robust standard errors.

^c^Not applicable.

**Figure 3:**
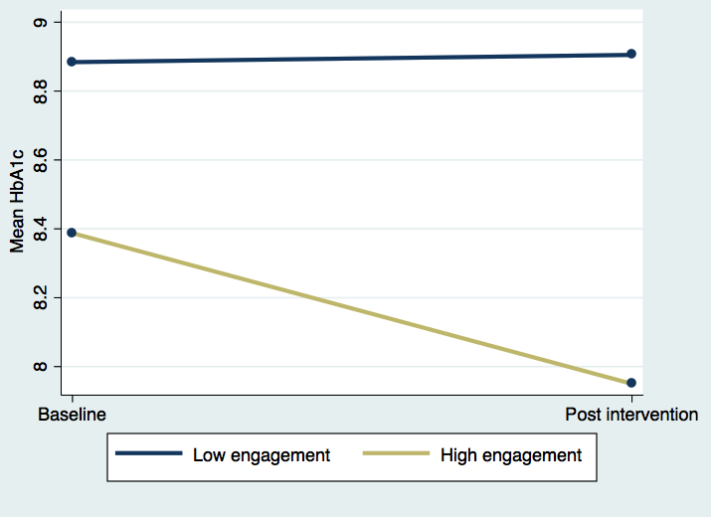
Change in unadjusted mean glycated hemoglobin (HbA_1c_) by patient engagement level. High engagement is defined here as having a response rate above or equal to the median of 64.5%.

Population-averaged linear models (Table 5) found that among the intervention participants, higher engagement (modeled through response rate to questions requiring a response) was associated with greater reductions in HbA_1c_, controlling for clinic site, age, gender, primary language, and race. In particular, highly engaged patients (defined as having a response rate ≥the median of 64.5%), experienced a mean 2.23 point reduction in HbA_1c_ relative to less-engaged patients (response rate <64.5%), controlling for demographics (*P*<.001; Model A). To illustrate the relationship between patient engagement and HbA_1c_, Figure 3 shows the changes in unadjusted mean HbA_1c_ values between highly engaged and less-engaged patients. As a sensitivity test, a population-averaged linear model was also run with a continuous, standardized response rate variable (Model B). This model found that an increase of 1 SD in response rate over the mean was associated with a mean 0.93 point reduction in HbA_1c_, controlling for demographics (*P*=.001), again supporting the findings that higher engagement was associated with greater reductions in HbA_1c_. Subsequent sensitivity analyses were also run using the lower and upper quartiles of engagement as cutoff points.

**Table 5:** Associations between patient characteristics and glycated hemoglobin.

Variable			Estimate	SE^a^	*P* value
**Model A with categorical response rate variable**					
	**Clinic**				
		Site 1	0.25	0.50	.62
		Site 2	Reference	Reference	Reference
	**Age, in years**				
		18-44	Reference	Reference	Reference
		45-54	1.38	0.57	.02
		55-64	−0.45	0.49	.36
	**Gender**				
		Female	Reference	Reference	Reference
		Male	−1.72	0.55	.002
	**Primary language**				
		Spanish	Reference	Reference	Reference
		English	2.05	0.73	.005
	**Race/ethnicity**				
		White	Reference	Reference	Reference
		Hispanic/Latino	−1.14	1.79	.52
		Other	−2.43	1.65	.14
	**Engagement with program**				
		Low (response rate <64.5%)	Reference	Reference	Reference
		High (response rate ≥64.5%)	−2.23	0.56	<.001
	Constant		10.72	1.80	<.001
**Model B with continuous, standardized response rate variable**					
	**Clinic**				
		Site 1	0.22	0.51	.66
		Site 2	Reference	Reference	Reference
	**Age, in years**				
		18-44	Reference	Reference	Reference
		45-54	1.21	0.64	.06
		55-64	−0.91	0.53	.09
	**Gender**				
		Female	Reference	Reference	Reference
		Male	−1.27	0.47	.007
	**Primary language**				
		Spanish	Reference	Reference	Reference
		English	1.72	0.62	.006
	**Race/ethnicity**				
		White	Reference	Reference	Reference
		Hispanic/Latino	−1.64	1.74	.35
		Other	−2.68	1.63	.10
	**Engagement with program**				
		Standardized response rate	−0.93	0.28	.001
	Constant		10.14	1.75	<.001

^a^Cluster-robust standard errors.

When defining highly engaged patients as those with a response rate above 32% (the bottom quartile), no statistically significant change in HbA_1c_ was found between highly engaged and less-engaged patients (results not shown in table). However, when defining highly engaged patients as those with a response rate above 86% (the top quartile), highly engaged patients experienced a mean 2.0 point reduction in HbA_1c_ relative to less-engaged patients (*P*=.001, results not shown in table).

Among intervention participants, being male was associated with a statistically significant decrease in HbA_1c_ relative to female participants, controlling for other demographic characteristics and patient engagement rate. In addition, speaking English as a primary language was associated with a statistically significant increase in HbA_1c_ relative to primarily Spanish- speaking participants, controlling for other demographics and response rate.

Table 6 presents findings on patient satisfaction with the text-messaging program. The overall response rate was 50%, due to substantial drop-off in responses as the text-message survey progressed to question 5. Among those who responded, satisfaction with the program was high: 78% (28/36) of respondents felt that they learned useful information from the text messages, and 89% (25/28) felt that the text messages helped them to better manage their diabetes. A post hoc regression model with cluster-robust SEs was run to examine any associations between satisfaction with the program and personal characteristics, including patient engagement among the intervention participants, but no statistically significant associations were found.

### Qualitative Results

#### Participants’ Feedback on the Program

Most participants (81%, 9/11) in the text-messaging program felt that the messages were positive. One participant stated:

...[the program was] positive, because it was telling us...what we have to do in our daily lives, and how a diabetic can’t be hopeless because it is a disease that can be controlled.

**Table 6:** Intervention group satisfaction with text-messaging program.

Statements and responses		n (%)
**I learn useful information from the text messages (n=36)**		
	Strongly agree/agree	28 (56)
	Not sure	1 (2)
	Disagree/strongly disagree	7 (14)
**I find the text messages annoying (n=29)**		
	Strongly agree/agree	6 (12)
	Not sure	4 (8)
	Disagree/strongly disagree	19 (38)
**The text messages help me better manage my diabetes (n=28)**		
	Strongly agree/agree	25 (50)
	Not sure	1 (2)
	Disagree/strongly disagree	2 (4)
**The text messages are clear and easy to understand (n=27)**		
	Strongly agree/agree	25 (50)
	Not sure	2 (4)
	Disagree/strongly disagree	0 (0)
**I would recommend the texting program to a friend with diabetes (n=25)**		
	Strongly agree/agree	23 (46)
	Not sure	2 (4)
	Disagree/strongly disagree	0 (0)

In addition, several participants explained that the program made them feel supported. This theme was especially common among Spanish-speaking participants (66%, 4/6). For example, 1 participant said:

The messages were helping me because these messages were as [if] a person was speaking to me, telling me what I should do, as if that message was from someone that was thinking of me and was telling me that I have to do this for my wellbeing.

Another participant said:

It felt good...because I knew that someone was worrying about my health.

In addition to emotional support, all participants (n=11) cited learning new information and setting new goals as a result of the program. Some participants felt the messages provided more detailed information than they get in medical appointments, and the text message format allowed them to refer back to the information. One participant said:

It’s just that the messages explains things...better. Because when I go to an appointment and ask, then the doctors speak in English and if the girls that they provide interpret for you, [they] don’t fully explain the conversation that you would have with a doctor.

Most participants also stated that they already knew some of the information (90%, 10/11), but many participants also struggled to recall specific content from the messages (63%, 7/11), suggesting that knowledge retention from the messages may be low.

Many felt that the messages provided helpful reminders (63%, 7/11) to check their blood sugar and/or to take their medication. All participants stated that the program led them to set new goals; to contemplate behavior change; or to change their behavior relating to their diet, medication, and/or exercise. For example, 1 participant reported taking their medication more regularly after the messages:

[The messages] said that you’re supposed to take [medication] twice a day at about the same time, and so we instituted a little thing where I have the little days of the week [on a]...holder that says, “Noon, Morning, Evening, Night,” and we put the pills in there so I take them on the right times...I’m doing it after the messages.

Some participants offered feedback to improve the program. A total of 2 participants felt that the times the messages were sent were not always convenient for them. Most participants wanted more messages, and 2 participants felt it would be helpful to tailor the program to participants’ baseline diabetes self-management knowledge levels. Additional quotes from the interviews with participants organized by theme are provided in Multimedia Appendix 2 for interested readers.

#### Implementing Staff Feedback on the Program

Staff who implemented the program identified key facilitators and barriers to the program’s success. The major facilitator cited by the staff was that this text-messaging program allowed them to provide health education to patients using relatively few resources, making implementation more feasible for a resource-limited FQHC. However, they also identified some barriers to program success, particularly for scale-up beyond the initial implementation for this effectiveness-implementation study. The clinic administration chose to use temporary staff (AmeriCorps volunteers) to enroll participants for this pilot, which minimized the program’s disruption to the clinic workflow but also limited integration into routine clinical practice. Interviewees suggested that no staff outside of those directly involved in management or enrollment (ie, none of the clinical providers) knew about the program. In addition, there was no systematic monitoring of patient responses, in part, because the text-messaging platform was not integrated with the electronic medical record system in the clinic. Similarly, identifying patients with diabetes eligible for the intervention was a challenge, requiring the clinic staff to print lists of eligible patients, cross-check them with the clinic schedule, and to identify patients when they presented for appointments. Much of the work to identify patients was done by the AmeriCorps volunteers, but these activities would likely be burdensome for permanent clinic staff if the program were to be scaled up to more patients with diabetes in the future.

Finally, staff also provided some feedback to improve the program in the future. A total of 2 staff members suggested that including more clinical staff could improve the program. One suggested that having clinicians mention the text messages during visits could give the program more “standing“ with patients. Staff also suggested hosting an in-person meeting at the start of the program to ensure all involved staff understand the project and their roles.

Overall, despite some of implementation barriers cited by staff, most felt the program worked well and had the potential to help patients with diabetes; some felt the program provided an easier-to-understand and more accessible form of health education than the brochures or written materials usually provided by FQHCs.

## Discussion

### Principal Findings and Comparison With Prior Findings

Participants of a diabetes text-messaging program described the program as providing instrumental and emotional support, and higher engagement with the program was associated with improvements in HbA_1c_. Earlier studies have found evidence of reductions in HbA_1c_ in broader populations who received a text-messaging intervention [5-7]. A recent randomized controlled trial of a text-messaging program in a similar low-income, Latino, diabetic population also found evidence of improved glycemic control following participation, though the program also collected patient-reported glucose levels via text message, unlike the CareMessage program [8]. We also examined BMI and BP, but no significant improvements were observed. This could be due to the relatively short duration of the study and/or the intervention’s emphasis on glycemic control for diabetes, rather than weight loss or BP specifically. Our findings suggest that text-messaging interventions for diabetes management might be effective among low-income Latino patients, if adapted appropriately. This finding is especially relevant given that earlier studies have found that these groups can have lower engagement with text-messaging programs and smaller health effects than other patient groups. We also found evidence that patients who are more engaged with the program might experience greater improvements to HbA_1c_, suggesting that encouraging patient participation could lead to greater health effects more broadly.

These findings indicate that this diabetes-management text-messaging program has the potential to improve HbA_1c_. The effect sizes seen in this study have potential to be clinically meaningful based on earlier studies. A meta-analysis of 5 earlier randomized controlled trials reported that a mean 0.9 point reduction in HbA_1c_ significantly reduced events of nonfatal myocardial infarction by 17% and events of coronary heart disease by 15% [20]. Therefore, applying these estimates to our findings, a mean improvement of 0.4 points (from the individual fixed-effects models, Table 4) could result in up to an 8% reduction in nonfatal myocardial infarction and a 7% reduction in coronary heart disease events. Among highly engaged participants, these effects could be even larger, where a mean reduction of 2.2 points in HbA_1c_ (from population-averaged linear models, Table 5) could result in up to a 40.8% reduction in nonfatal myocardial infarction and a 36% reduction in coronary heart disease events.

Qualitative analyses highlight the potential mechanisms that could lead to improved intermediate outcomes for people with diabetes participating in the program. Many participants cited receiving both instrumental and emotional support from the program. First, participants described how the messages reminded them to take their medication or to check their blood sugar. These descriptions evoked “cues to action” as described by the Health Belief Model and found by other studies of similar interventions [14]. Though the constructs of this model were not assessed directly in this study, the CareMessage text-messaging platform was informed by the Health Belief Model, and patient interviews explored these concepts. Then, participants also described feeling that someone was thinking or worrying about them, suggesting that they received emotional support from reading and responding to the messages, particularly among Spanish-speaking participants. These results aligned with earlier findings that text messages for diabetes management were able to produce greater positive and optimistic feelings in patients as well as reducing denial of diabetes among patients participating in these types of programs [11]. Similar findings have also been observed among Spanish-speakers in a text-messaging intervention for depression [21].

The interviews of patients and staff identified some facilitators and barriers to the implementation of this program. The ease of reaching many patients at once with diabetes self-management information made this program significantly more feasible for a resource-limited FQHC. However, the clinic experienced challenges of integrating the program into their routine care processes. Recommendations to facilitate implementation and improve patient experiences include adapting the messages to baseline patient knowledge and linking in-person clinical care with the text-messaging program. These types of improvements could have positive effects on patients’ satisfaction with the program as well as patients’ engagement with the program, which could lead to improved self-management and outcomes of care, but they would also require changes in provider behavior and clinical workflow.

### Limitations

This study has important limitations. First, because the text messages were implemented for this pilot study within the participating clinics’ constraints, the analytic sample is modest. The comparison group patients were also not aware of the intervention or the analysis of their deidentified data, so there is a likelihood that any observed improvements to intervention participants’ HbA_1c_ could have been due to the Hawthorne effect. In addition, operational constraints were not conducive to randomizing patients to the intervention and comparison groups, which could have improved causal inference. As a result of the lack of randomization, we cannot conclusively determine that the intervention caused any observed differences between the groups. However, we were able to use propensity score weighting to balance confounding factors between groups, reducing concerns about selection bias. A second limitation of this study is missing data. Despite the use of a long observation period following the intervention (1 year), about 22% of the intervention group did not attend a follow-up visit in that period, leading to missing outcome data. However, when comparing the baseline HbA_1c_ of patients who came for a follow-up visit with those who did not, we found no evidence of a statistically significant difference in HbA_1c_ among nonreturning patients, reducing concerns about bias. If the long follow-up period had any effect on the results, it would have had an attenuated effect on the intervention group’s outcomes, biasing our results toward the null. A third limitation to this study is that the qualitative interviews were only conducted with patients who volunteered to participate and therefore, might not be representative of all patients’ experiences with the program. Interviewed patients, however, provided critical feedback to improve the program. Another important limitation is that the follow-up patient satisfaction questions and diabetes-related distress (PAID-5) had low rates of response, likely due to the delivery via text message late in the program and the large number of questions delivered. In the future, response rates could potentially be improved by delivering this survey in person during a visit to the clinic (as was done with the PAID-5 measure at baseline) or by incentivizing completion. Finally, we do not have data on the proportion of messages actually received and read by participants, and there is a possibility that mobile phone plans or changes to phone numbers could have affected receipt of the messages. However, 100% of the messages were reported as delivered by the text-messaging platform, and 86% of participants responded to at least 1 question with a valid answer, suggesting that if there were patients who did not receive the messages, it was not a widespread issue.

### Conclusions

This study contributes to our understanding of the effectiveness of diabetes management text-messaging programs among patients who have low income and are mostly Latino. We found evidence that glycemic control of adult patients of FQHCs with diabetes might be improved through participation in a text-messaging program for diabetes self-management. The findings also suggest that patient engagement with the program could contribute to improved self-management and clinical outcomes. By supporting patients with education, reminders, and positive messages during the course of their daily life, diabetes management text-messaging programs have the potential to increase and sustain healthy behaviors and improve clinical outcomes among low-income patients with diabetes.

## Appendix

Multimedia Appendix 1 English versions of the interview guides used for patient and staff qualitative data collection.

Multimedia Appendix 2 Additional quotes from the qualitative interviews, organized by theme.

## Abbreviations

BMI: body mass index
BP: blood pressure
FQHC: federally qualified health center
HbA_1c_: glycated hemoglobin
PAID-5: Problem Areas in Diabetes questionnaire (short form with 5 questions)
