# Supplementary material for: Improved Diabetes Care Management Through a Text-Message Intervention for Low-Income Patients: Mixed-Methods Pilot Study
Source: JMIR Diabetes. 2018 Oct 30;3(4):e15. doi: 10.2196/diabetes.8645 (PMC6238849; doi:10.2196/diabetes.8645)
Supplement: Multimedia Appendix 1 [file diabetes_v3i4e15_app1.pdf]

Patient Participant Interview Questions

1. Who enrolled you in the program? Did you find that getting signed up was easy?
2. Describe your first encounter with the text messages. What did you think?
3. Who did you think the messages were coming from?
4. Do you think the number of messages (e.g., should the frequency of messages be customizable by patient) was too few, about right, or too many? Why?
5. Were the education messages about diabetes helpful? Please tell me a couple of key things you remember from the messages.
6. Were the messages positive, neutral or serious? How would you describe the tone of the program?
7. After using CareMessage and thinking more about your diabetes, did you set any new goals for improving your diabetes control? What were your goals and did you feel that you made progress?
8. To what extent did the messages impact your exercise, smoking, medication taking, and/or diet?
9. Was the system an effective way to get answers to your questions?
10. Did you discuss the messages with any of your family or friends?

Implementing Staff Interview Questions

1. Can you describe how your clinic is going about planning and implementing the CareMessage program? What is your role?

2. *[RELEVANT STAFF ONLY]* Are you comfortable explaining what CareMessage is to patients? Do you feel like the patients understand the concept of the text-messaging program?

3. How easy or difficult has it been to incorporate CareMessage into your workflow? How much did the program change your interaction with patients? Describe your new routine. On a scale from 1 to 10, how disruptive was the addition of CareMessage into your existing workflow (1 being not disruptive and 10 being very disruptive)? What was disruptive about the implementation?

4. How many people in the clinic have been affected by the implementation of CareMessage?

5. Have you had any training or resources (protected time, etc.) for implementing the CareMessage program? If so, probe: What are these resources? What other resources or supports do you think might help with implementing the change?

6. Do other clinicians and staff not directly involved in the implementation of the program know about the changes? If so, probe: How are they reacting to these changes?

7. What is working well in this program?

8. What challenges have you experienced so far with this program?

9. Do you monitor patient responses in the program? If no, why not? If so, what do you monitor and how does it impact the care the patient receives?
10. Is there anything else that you can think of that may help you and other staff as you implement this change? Anything else that is hindering or creating barriers for implementing this change?
