# Supplementary material for: Improved Diabetes Care Management Through a Text-Message Intervention for Low-Income Patients: Mixed-Methods Pilot Study
Source: JMIR Diabetes. 2018 Oct 30;3(4):e15. doi: 10.2196/diabetes.8645 (PMC6238849; doi:10.2196/diabetes.8645)
Supplement: Multimedia Appendix 2 [file diabetes_v3i4e15_app2.pdf]

Example Quotes from Qualitative Interviews with Intervention Participants (n=11)

Theme	Quote
Felt supported (5/11)	"The messages were helping me because these messages were as a person was speaking to me, telling me what I should do, as if that message was from someone that was thinking of me and was telling me that I have to do this for my wellbeing."
	"It was really encouraging, I thought, because if you, for example, asked how I felt about diabetes after I was diagnosed, is it helping me to manage it better, I mean that was some of the multiple choices. And let's say if I answered that, you know, I was depressed, whatever, then it would send me like a really encouraging message that, you know, hey diabetes can, you know, because of diabetes you'll learn to live a more healthy life, you know, something positive that I thought."
	"It felt good also because I knew that someone was worrying about my health."
	"I thought that there was people that think about your life more than anything else. That's what I liked. That they are trying to help you so that you don't like with the mindsight of "Oh, I am going to die or this is going to happen to me." You understand? To know what you need to do in order to look for a solution, not to be stuck on something that you know you already will have. And to not think negatively."
Learned new information (11/11)	"It's just that the messages explains things more… Better. Because when I go to an appointment and ask, then the doctors speak in English and if the girls that they provide interpret for you don't fully explain the conversation that you would have with a doctor."
	"Ah, yes. Because the medical staff only prescribe me things, they don't advise. They should advise the patient more and I did feel that the message that I received was more helpful in terms of informing more about diabetes."
	"Oh wow did you know that, hey I didn't know this," it's something new, new things that are in there."
	"I had no idea, you know, how bad carbs are for you. Now I read the labels."
Set new goals, contemplated behavior change, or changed behavior (11/11)	"First off, I am more calm. In another aspect, because I am checking my sugar levels and it is lower and I am doing things that I did not do before, such as doing exercise, having tranquility, be more positive for myself, taking myself into account, appreciating myself as a person and taking my medication how they are supposed to because I wouldn't take them how I was supposed to before."
	"So I have had my daughter in law make me up a sheet of papers to print out where it has that I took it in the morning and I pinned it on the wall… going in and out of my room I see this… I took this insulin and then I just mark it and I put the time. So that helped me, I'm not gonna say it helped me to do that but I would say it kind of inspired me to pay more attention to my diabetes."

	“Before I didn’t take it into consideration and would continue as if I didn’t have diabetes. It never impacted me, and I never took into consideration to keep taking care of myself. That is why it helped me change the way I would live and eat.”
	“Because you know, I was reminded by the text, so I get out walking again. You know, not that far, because I got tired. But then I began walking more. So now I walk about an hour a day.”
	“Sometimes I do the chores later in the day and in the radio they put on cumbias and I will be sweeping and mopping and then I finish after an hour because that’s how long they put the songs on for. At times there are resting periods and I dance one cumbia and then the other, like two or three songs I continuously dance to… Because I have the broom and it seems like it is a partner and you dance and at the same time you are exercising right? Even though it’s a little bit, but it works, everything counts.”
	“It said that you're supposed to take it twice a day at about the same time, and so we instituted a little thing where I have the little days of the week and I have a little... holder that says, you know, "Noon, Morning, Evening, Night," and that, we put the pills in there so I take them on the right times… I'm doing it after the messages.”
	“Well look, I ate a lot of bread. I ate a lot of bread and tortillas. That were some of my excessives. That was the reason why I couldn’t lose weight because honestly I would say. “No it is a little bit, two in the morning, three in the afternoon.” It was five tortillas at times… So when the messages would say that everything that was carbohydrates would turn into sugar, I would say, well then imagine, five tortillas that at times I eat… My body doesn’t want to eliminate it, and if it can’t eliminate it, well I shouldn’t be putting into the body what it can’t eliminate right? And well I did do some changes. Right now what is bread and tortillas I don’t eat anymore. Not even one tortilla, it’s been like three months that I don’t even eat one tortilla. I only use the fork. Before I wouldn’t do that and I am feeling better. I am losing weight.”
Helpful reminders (7/11)	“I felt that these little messages that I got daily kind of reminded me to take my insulin and take my medication. Because I may have been-- left the house and then all of a sudden I get this ding! And I’m like oh man and then I look and then it’s like, “Oh I forgot to take my pills.”
	“Yeah, I think the way they did it was fine because it’d kinda catch me off guard and then it would remind me to do these things. ‘Cause sometimes I get in a rush and then I’ll forget about checking my blood or I’ll-- proper stuff to be eaten and to-- it was a constantly reminder of things that I needed to do and things I should be aware of. So I thought that was pretty cool ‘cause then-- because I get caught up and I forget I even have diabetes and stuff.”
